# Hepatitis C Prevalence on the Rise but Screening at Safety Net Institutions Lagging behind

**DOI:** 10.1155/2023/3650746

**Published:** 2023-11-08

**Authors:** Jarin Prasa, Syed S. Karim, Bobby Jacob, Paul Mustacchia

**Affiliations:** ^1^Department of Internal Medicine, Staten Island University Hospital, NY, USA; ^2^Department of Gastroenterology, NYC Health and Hospitals, South Brooklyn Health, NY, USA; ^3^Department of Gastroenterology, Parkview Regional Medical Center, IN, USA; ^4^Department of Internal Medicine and Department of Gastroenterology, Nassau University Medical Center, NY, USA

## Abstract

**Introduction:**

In the United States, the hepatitis C virus (HCV) is a leading contributor to liver-related illnesses and fatalities. Despite effective antiviral medications, acute infections have increased in recent years, likely due to IV drug use and the opioid epidemic. Previous guidelines recommended one-time screening for individuals born between 1945 and 1965. The CDC now recommends screening all adults over 18 unless there is a low prevalence in the area. Accurate measurement of HCV prevalence is essential for targeted prevention. In New York, over 100,000 individuals have HCV. We present data on HCV screening at a safety net hospital in Long Island, NY.

**Objective:**

To identify screening rates for hepatitis C and the exposure prevalence and specific demographics of a community in Long Island, NY.

**Methods:**

We performed a review of all patients seen in our hospital from 2012 to 2019. We identified patients born in the years 1945 to 1965 using our electronic medical record (EMR) system and subsequently analyzed those who were anti-HCV positive. We reviewed their demographics, including age, gender, and ethnicity, as well as their history of intravenous drug use and HIV coinfection status. Basic statistical analysis was used.

**Results:**

Our study identified 21,722 patients born between 1945 and 1965 and found that only 8.5% or 1,858 individuals were screened for hepatitis C. Among them, we found that 5.9% (109) tested positive for HCV antibody, with 3.0% (56) having an active infection. Demographic characteristics of those with HCV antibodies included 70.6% male, 53.2% Caucasian, 33.9% Black, and 15.6% persons who inject drugs (PWID).

**Conclusion:**

Our study findings suggest that a significant portion of patients in our community had missed opportunities for screening in our hospital. Our community had an estimated 5.9% prevalence, higher than the national and state averages. Caucasian men had higher prevalences. This study suggests the need for broader screening initiatives and more focused resource allocation, perhaps to safety net institutions, to decrease the burden of HCV.

## 1. Introduction

Approximately 4.1 million people have hepatitis C exposure in the US [1]. 55 to 85% of patients infected with HCV develop chronic liver disease and 15 to 30% develop cirrhosis [2]. Hepatitis C has historically been the leading cause of chronic liver disease and hepatocellular cancer, leading to liver transplantation in the US [3, 4]. Only recently, with data from 2010 to 2019, nonalcoholic fatty liver disease and alcohol-related liver disease have surpassed HCV as the leading cause of liver transplantation, likely due to advances in antivirals and possibly due to increases in metabolic syndrome [4]. However, despite new antiviral medications that have near 100% success rates, acute infections from hepatitis C have increased over the last decade, according to data from the CDC [5]. IV drug use is one of the greatest risk factors for acquiring the infection, and the dramatic increase in the heroin and opioid epidemic may have contributed to the increase in hepatitis C infections [6]. Previously the United States Preventive Service Task Force (USPSTF) and the CDC recommended screening the baby boomer population, those born between 1945 and 1965, as they were noted in previous data to carry the greatest burden of hepatitis C. However, recently, the CDC screening recommendations have changed to reflect the increasing prevalence to any adult above 18 unless there is an extremely low prevalence in the region. Accurate measurement of the prevalence of HCV is crucial to implementing strategies for targeted populations. These strategies can include improving screening modalities, outreach programs to reduce risk factors for hepatitis C, such as IV drug use, and connecting individuals to physicians for tailored treatment plans. New York is among a few states that have more than 100,000 individuals with HCV [7]. Due to missing prevalence data, a mathematical model developed by the University of Albany estimated that 189,000 individuals have HCV in NY [8]. Concerningly, there is limited data on HCV exposure prevalence in local communities, raising concerns if effective screening strategies are in place. We present the prevalence and demographics of HCV-exposed individuals screened in a safety net hospital in Long Island, NY.

## 2. Methods

The study was conducted at our institution, which is a 530-bed level 1 trauma center. It is located in a suburban area of Long Island, New York, with a diverse patient population that includes urban, suburban, and rural areas. Our institution, like other safety net hospitals, provides care to a large number of uninsured, underinsured, and low-income patients. The hospital provides care to a wide range of patients, including those with acute and chronic medical conditions, mental health disorders, and substance use disorders.

Since 2012, our institution has attempted to screen for HCV in patients born within the previous screening age cohort. Patients who were born between 1945 and 1965 who were seen in various departments of the hospital were flagged in the EMR system for the provider to order an HCV antibody test with subsequent reflex RNA testing if the HCV antibody was positive. This automatic EMR flag was present regardless of the type of patient encounter, and this included outpatient medical clinics, surgical clinics, the emergency department, inpatient hospitalizations, and even psychiatric encounters.

In our study, we reviewed data from January 1, 2012, to August 31, 2019, specifically focusing on individuals born between the years 1945 and 1965. To gather data, we utilized our EMR system to identify patients who were positive (+) for HCV antibodies. Our biostatistics department used our EMR and gathered the total number of patients within the age cohort seen in our hospital and the total number of patients actually tested for hepatitis C with HCV antibody testing along with the data for HCV RNA from reflex testing. For the individuals who were positive for HCV antibody, their medical record numbers were separately given to us for review. From there, we conducted a thorough retrospective chart review, taking into consideration various demographic factors such as age, gender, and ethnicity, as well as whether or not the individual was a person who injects drugs (PWID) and their HIV-coinfected status.

Demographic information was extracted from certain sections within the EMR, such as the face sheet. PWID status was found by thoroughly reviewing the physician notes describing the social history. HIV coinfection, hepatitis C treatment status, and end result were extrapolated from reviewing the patients' lab results. Basic statistical analysis was then applied to the data collected. This comprehensive approach allowed us to gain a more well-rounded understanding of the HCV prevalence in our patient population.

## 3. Results

**Figure 1:**
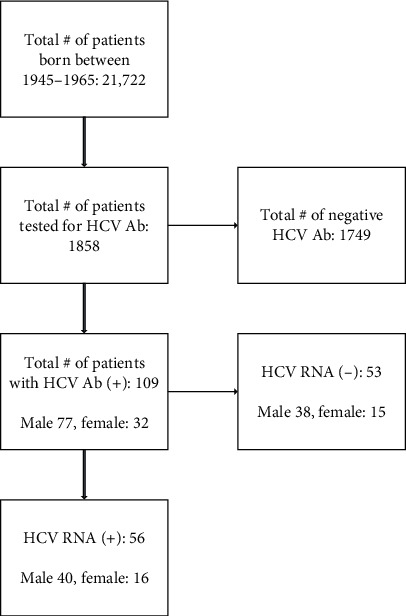
HCV antibody screening for patients born between 1945 and 1965 during January 1, 2012, to August 31, 2019.

Our study analyzed data from 21,722 patients between January 1, 2012, and August 31, 2019, who were born between 1945 and 1965.  Figure 1 shows that of the total number of patients seen in our hospital, 1,858 individuals (8.5%) were tested for HCV antibodies, and 109 (5.9%) of them tested positive. All 109 of the patients who were found to have HCV antibodies had automatically been tested for HCV RNA. Further testing revealed that 56 (3.0%) of these patients had active HCV infection with detectable RNA. We also examined the demographic characteristics of those who tested positive for HCV antibodies, as shown in Table 1. Of the 109 individuals who tested positive, 77 (70.6%) were male, 32 (29.4%) were female, 58 (53.2%) were Caucasian, 37 (33.9%) were Black, and 6 (5.5%) were Asian. We also looked at other factors related to hepatitis C infection. Of those who tested positive for HCV antibodies, 17 (15.6%) had a history of intravenous drug use (PWID), 4 (3.7%) had HIV coinfection, 5 (4.6%) had received previous treatment for HCV, and 2 (1.8%) had achieved sustained virologic response (SVR).

## 4. Discussion

The National Health and Nutrition Examination Survey (NHANES) data, which is a representation of the noninstitutionalized population in the US, has historically shown that a majority of individuals burdened with HCV were born between 1945 and 1965 [1]. This finding led to a shift in HCV screening recommendations by both the CDC and USPSTF, who had recommended one-time screening for individuals born within that time frame instead of only screening those with risk factors and symptoms. NHANES data from 2003 to 2010 revealed that about 3.6 million individuals in the US have hepatitis C exposure by evidence of hepatitis C antibodies [9]. According to NHANES 2010 data, the national average of anti-HCV is 1.67 to 2% in the US [7]; however, this number is an underestimation since NHANES only accounts for noninstitutionalized individuals, leaving out millions of individuals in jails, hospitals, or those who are homeless [10]. Several studies have reported HCV exposure prevalence by measuring antibodies to hepatitis C. State-level statistics and demographics revealed that the Western states have the highest prevalence. In the Northeast, New York is lower than the national average but still has over 100,000 individuals with anti-HCV [7]. Unfortunately, limited data exists for local communities within each state for anti-HCV prevalence. In our study, we estimated the prevalence of HCV antibodies in our community, which was found to be 5.9%, much higher than the national average of 1.67 to 2% and even that of our state, New York, at 1.49% [5]. Men were more likely to be exposed than women, correlating with the male-to-female HCV prevalence ratio of 2.3 found nationally by Bradley et al. [11]. Our hospital is a safety net hospital located in Long Island, NY, where the high prevalence in our community can be explained by the heroin and opioid epidemic in the Long Island area [12]. Intravenous drug use is a great risk factor for HCV, and although only 15.6% of individuals with anti-HCV had a history of IV drug use, our study was limited since this was a retrospective study and patients are not extensively screened for drug use during routine history taking, and the patient usually volunteers this information to their providers.

Only a mere 8.5% of patients who met the birth year criteria for hepatitis C screening, as per the previous guidelines, were screened at our hospital. This is a concerning trend as recent data from the USPSTF suggests that community health centers across the nation are also experiencing similarly low screening rates, with only 8.3% of patients being screened [1]. In fact, an analysis of four safety net institutions in underserved areas revealed a screening rate of only 0.8% [1]. It is important to note that the patient population analyzed in our study included those seen in various settings, such as the emergency room, psychiatric units, inpatient hospitalizations, and outpatient clinics. However, screening practices typically occur during primary care encounters, which may have contributed to the low screening rate. Future research can take into account individualized data from each of these departments to verify this theory. Another potential explanation for the low screening rate is the lack of awareness regarding the previous screening guidelines, which targeted a specific group within a particular birth year. The updated, simplified recommendation may help increase awareness and improve screening rates. Additionally, it is noteworthy to state that although there was a low uptake of screening, there was a high prevalence of the disease, and this may be due to a case-finding strategy instead of pure screening strategies implemented by the providers not knowingly. Although there was a formal screening strategy in place by the institution by means of EMR prompts, providers may have actually screened individuals who were determined to be high risk or who had elevated liver enzymes, which would explain the low screening rate further.

This study highlights the need for broader screening initiatives and more focused resource allocation to decrease the burden of HCV in our community and likely that of other safety net institutions. National guidelines provided by organizations such as the CDC and USPSTF need to be matched to the local population served, and public health programs should resource communities with a high HCV burden to most effectively diagnose and treat individuals actively infected with HCV before the development of chronic liver disease. However, there are potential local efforts with minimal resource allocation that can be implemented while advocating for more resources. Cost-effective strategies include educating the high-risk population, such as PWID, about the importance of screening and the availability of affordable treatment. Community campaigns and outreach programs can be organized to increase awareness and encourage individuals to get tested. Healthcare providers should be educated about the current guidelines regarding screening all adults and be reminded to screen via EMR prompts. These efforts can be a starting place to improve screening modalities. Ultimately, a combination of increasing screening efforts in addition to the focused allocation of resources, aimed explicitly at safety net institutions, may be an effective strategy for decreasing the HCV burden and its sequela in the population.

The lessons learned from screening baby boomers for HCV are especially relevant now as we have an even larger population in need of screening. With the new guidelines recommending screening for all adults, there is a pressing need to improve screening modalities to ensure that all individuals are screened for HCV. The low screening rates observed in the baby boomer population highlight the need for increased education and awareness among healthcare providers and patients to ensure that screening is conducted regularly. This is especially important given that HCV can be asymptomatic for years, and many individuals may not be aware that they are infected. Identifying individuals with HCV early through improved screening modalities can lead to better outcomes and reduced transmission rates. Therefore, it is crucial to apply the lessons learned from screening baby boomers to ensure that all adults are screened for HCV, regardless of their age or risk factors.

## Figures and Tables

**Table 1:** Demographic data of patients screened and found to have HCV Ab.

Demographic	HCV Ab (+)
Gender	
Male	77 (70.64%)
Female	32 (29.36%)
Ethnicity	
Caucasian	58 (53.21%)
Black	37 (33.94%)
Asian	6 (5.50%)
Unknown	8 (7.34%)
HIV coinfected	4 (3.67%)
PWID^∗^	17 (15.60%)
Treatment experienced	5 (4.59%)
With SVR^∗^	2 (1.83%)
No SVR	3 (2.75%)
Total	109

^∗^PWID: persons who inject drugs; SVR: sustained virologic response.

## Data Availability

The data used to support the findings of this study are included within the article.
